# Unravelling Dynamics Involving Multiple Charge Carriers in Semiconductor Nanocrystals

**DOI:** 10.3390/nano13091579

**Published:** 2023-05-08

**Authors:** Krishan Kumar, Maria Wächtler

**Affiliations:** 1 Department Functional Interfaces, Leibniz Institute of Photonic Technology Jena, Albert-Einstein-Straße 9, 07745 Jena, Germany; 2 Chemistry Department and State Research Center OPTIMAS, RPTU Kaiserslautern-Landau, Erwin-Schrödinger-Str. 52, 67663 Kaiserslautern, Germany

**Keywords:** nanorods, transient absorption spectroscopy, charge carrier dynamics, multiple excitons, photocatalysis

## Abstract

The use of colloidal nanocrystals as part of artificial photosynthetic systems has recently gained significant attention, owing to their strong light absorption and highly reproducible, tunable electronic and optical properties. The complete photocatalytic conversion of water to its components is yet to be achieved in a practically suitable and commercially viable manner. To complete this challenging task, we are required to fully understand the mechanistic aspects of the underlying light-driven processes involving not just single charge carriers but also multiple charge carriers in detail. This review focuses on recent progress in understanding charge carrier dynamics in semiconductor nanocrystals and the influence of various parameters such as dimension, composition, and cocatalysts. Transient absorption spectroscopic studies involving single and multiple charge carriers, and the challenges associated with the need for accumulation of multiple charge carriers to drive the targeted chemical reactions, are discussed.

## 1. Introduction

Semiconductor nanocrystals have been extensively studied for their excellent size-tunable optoelectronic properties in the past few decades [1,2,3]. These nanocrystals have been envisioned to be useful in applications such as light-emitting devices, field-effect transistors, solar cells, light driven catalysis, infrared detectors, lasing, etc. [3,4,5,6,7]. The application of semiconductor nanocrystals in direct solar-to-fuel conversion is particularly interesting, transforming the sunlight’s energy into chemical energy by forming highly energetic molecules, e.g., hydrogen from water [8,9], or methanol [10], methane [11,12], or carbon mono-oxide [13] from CO_2_. In a typical solar-to-fuel conversion device, photons of suitable energy are absorbed by the nanocrystal, generating charge carriers which migrate to the catalytic reaction site where they are consumed, driving a redox reaction, e.g., proton reduction. To carry out photochemical water splitting, many parameters must be considered. Here, we only aim to name the main aspects that are important for the design of a photocatalyst. For a detailed understanding of these parameters, we refer the reader to review other articles in the field [8,14,15,16,17,18,19]. (1) Besides a band-gap delivering sufficient driving force, the band-edge positions of the semiconductor should be suited to support the photochemical reaction of interest, in order to ensure the practically reliable rate of photoconversion overpotential is required. (2) Often, a cocatalyst is used in this regard to maintain the overpotential as sufficiently low and to speed up the reaction. (3) The lifetime of excited charge carriers generated by photoexcitation should be on a similar order of magnitude as the photochemical reaction [20]. This means that the charge separating interface, e.g., between the nanocrystal and cocatalyst, must be designed to support efficient charge separation and prevent or minimize charge carrier recombination. (4) Furthermore, the photocatalyst should be stable under continuous illumination.

The energy required to drive the overall water splitting reaction is about 1.23 eV [14]. This means that the band-gap of a photocatalytic system consisting of just one material must be larger than this value or a Z-Scheme approach must be followed, combining several light-absorbing units by a shuttle redox mediator, allowing the use of lower energy photons [21]. For water splitting, chalcogenide-based nanocrystals are interesting, as they have, in principle, suitable band positions to drive the overall water splitting reaction, combined with high absorption cross-sections in the visible optical window, thereby efficiently absorbing a large portion of the sunlight to generate charge carriers. Further, their band-edge energy can be fine-tuned via their size and dimensions, exploiting quantum confinement effects to match the energetic demands of the targeted photocatalytic reaction [14,22]. Due to their low dimensions, charge migration distances to the interfaces and reaction sites are short, which reduces losses due to recombination and increases the lifetime of charge carriers. Further, by the design of heteronanostructures, band-gap engineering can be used to spatially separate the charge carriers to suppress recombination pathways [23,24]. Introducing additional cocatalysts can help to minimize the charge recombination by facilitating charge separation, besides reducing the activation energy barrier and, hence, improving the reaction rate for the catalytic reaction [25,26,27,28,29]. Typical cocatalysts applied for hydrogen evolution are molecular systems [30,31] or metallic clusters; e.g., Ni or Pt particles have almost negligible overpotential for proton reduction and can be used as electron sinks [25,26]. The photo-instability of cadmium-based chalcogenide arises mostly due to the presence of photogenerated holes, resulting in photooxidation [23,32], which limits long-term photocatalytic activity. To achieve photostability of the nanocrystals, many approaches have been undertaken, e.g., the photostability of the nanocrystal materials can be improved by tuning surface passivating ligands [33,34] or by the use of suitable shell material [23,35,36,37,38] to passivate trap states and prevent trapping of photogenerated holes in surface sites, leading to degradation via photooxidation. Also, the use of a suitable sacrificial donor to quench the photogenerated holes can enhance the photostability of the photosystem [39]. A final challenge is the fact that, to carry out a complete photocatalytic reaction, the accumulation of electrons is required, e.g., proton reduction to hydrogen requires 2e^−^, CO_2_ reduction requires 6e^−^ and 8e^−^ to methanol and methane, respectively. Besides the general challenges of multi-charge-carrier transfer, additionally, the availability of multiple acceptors can counteract charge-carrier accumulation at the active sites and lead to a decrease in catalytic efficiency [40,41,42,43,44]. It is very challenging and complicated to optimize a full redox cycle at once. Therefore, quite often, sacrificial agents are used to simplify the full cycle to one half-cycle and optimize the model systems for one half-reaction of artificial photosynthesis first [45,46]. However, the ultimate goal of photocatalysis is to have a self-sustaining system, including coupled half reactions [31], without relying on the use of sacrificial agents, which reduces the gain from photocatalysis.

**Figure 1:**
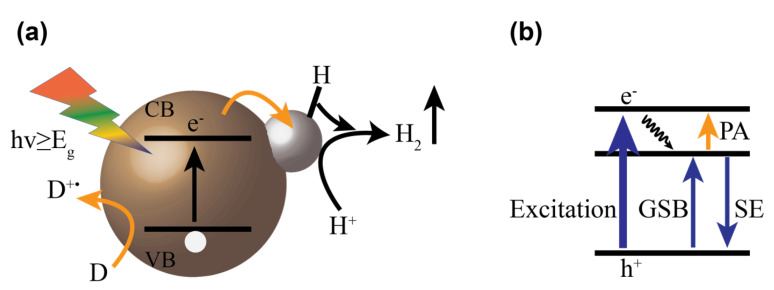
Schematic representation of photocatalytic hydrogen evolutions involving a sacrificial agent (**a**) and schematic to show origin of different signals in nanocrystals (**b**). GSB: Ground State Bleach, PA: Photoinduced Absorption, and SE: Stimulated Emission.

Hydrogen evolution through nanocrystals (see Figure 1a) has been shown using nanocrystals of varying dimensionality, i.e., 0D quantum dots, 1D nanorods, or 2D nanosheets [7,47,48,49,50]. The dimensions of the nanocrystals can have a strong impact on their properties. As an example, we discuss here the properties of rod-shaped particles. Compared to quantum dots, nanorods show higher absorption cross-sections [51,52]. Nanorods exhibit quantum confinement effects in the radial direction and bulk-like transport properties in the axial direction, which can support the fast long-range transfer of photogenerated charge carriers along the axis. Hydrogen production has been demonstrated using structures derived from the CdSe or CdS nanorods, which have been functionalized with nanoparticles selectively at the tips [37,53,54,55]. The dimensions and composition of the semiconductor nanocrystal strongly impact the activity of the systems. Increasing the length can have two consequences. First, it results in higher absorption cross-sections, leading to enhanced light absorption. Second, the generated charge carriers must travel a longer distance to reach the active site, which could result in decreased electron transfer rates to the catalytic site. A tradeoff between these opposing factors is discussed as they impact the optimum length of a Pt-tipped CdSe nanorod photocatalyst for hydrogen generation. Choi et al., report that single-tipped nanorods of 15–20 nm length show optimum hydrogen generation efficiency in a series of nanorods of varying length [42]. In contrast, CdS and derived dot-in-rod structures, e.g., CdSe@CdS nanorods, show increasing conversion efficiencies with increasing length [37]. Besides light absorption and charge separation, recombination must also be considered. Rod length increases potential distances between the holes remaining in the nanorods and the electrons transferred to the metal particles at the tip, leading to increasing lifetimes of the charge separation with increasing nanorod length. Especially in seeded nanorods, this defined functionalization in combination with the charge carrier localization within the semiconductor nanocrystal, determined by the band-edge offsets in the heterostructure and leading to hole localization in the seed, results in long range charge separation with extremely long lifetimes, supporting charge carrier accumulation at the reaction site and improved photon-to-hydrogen conversion efficiencies compared to the non-seeded system [56,57]. In seeded nanorods, additionally, the band alignment can be tuned from type I to quasi-type II using the seed size and/or shell thickness, often referred as “wave-functions engineering” [58,59]. The smaller size CdSe seed results in a quasi-type II band alignment, localizing photogenerated holes in the CdSe seed while delocalizing electrons over both domains, and a larger-sized seed results in type I band alignment with preferred hole and electron localization in the CdSe seed. Indeed, Amirav et al. have observed more than a doubled apparent quantum efficiency of hydrogen production with a change in seed size from 3.1 to 2.3 nm in dot-in-rod nanoheterostructures having a 3 nm platinum tip and a rod length of about 60 & 70 nm [37]. This observed increase in efficiency is presumed to be associated with higher degree of electron and hole separation in the smaller seed size of CdSe forming the quasi-type II band alignment, supporting the transfer of electrons to the metal tip.

The performance of the systems was shown to strongly depend on the reaction conditions, e.g., the sacrificial agent used depends strongly on efficient hole extraction. CdS-Pt with methanol as the hole scavenger showed a hydrogen generation efficiency below 1%, whereas, with sulfite hole scavenger, the efficiency shot up to ~10% in these systems, which positively correlates to the rate of hole quenching [39,55,60]. This observation could be related to the prevention of charge carrier recombination by efficient hole quenching on the one hand, and prolonging the stability of the nanostructure by preventing photodegradation on the other hand. The photon-to-hydrogen conversion efficiency is often limited by hole scavenging and the use of an additional hydroxyl anion-radical redox shuttle has been shown to efficiently remove the hole out of the photocatalyst and give a record 100% photon-to-hydrogen generation efficiency [56,61,62].

To summarize the discussion above: in the design of nanoparticulate photocatalysts, complex interplay between structural parameters which impact a delicate balance between single steps in the underlying mechanism must be considered to develop improved systems. The foundation for this approach is a detailed understanding of how dimensions, composition, surface properties, types of functionalization, etc., impact the single steps leading to the targeted function. For this, the charge carrier dynamics in these systems must be studied. Time-resolved spectroscopy, especially transient absorption spectroscopy (TAS), is a highly useful technique for studying the charge carrier dynamics in semiconductor-based photocatalysts. The use and interpretation of transient absorption spectroscopic data on semiconductor nanocrystals have been extensively described in the literature and will only briefly be summarized here [63,64,65]. Briefly, a short pump pulse of suitable energy excites the system, and a temporally delayed probe pulse is used to monitor the absorption properties in the excited system. The difference between the absorption spectrum of the excited system and the absorption spectra of the not-excited system (ΔA) is recorded as a function of the time delay between the pump and the probe pulse, allowing for monitoring the development of the system after excitation. The signal in TAS arises mainly from three factors: loss of ground state population, excited state absorption, and stimulated emission (see Figure 1b). The ground state bleach in semiconductor nanocrystals arises because the pump pulse excites part of the nanocrystal ensemble, generating electron hole pairs. Due to the population of conduction band states (and in principle also the depopulation of valence band states), the excited nanorods show a decreased absorption at their respective peak positions, resulting in a negative ΔA. In the context of II–VI nanocrystals, valence band states have higher degeneracy involving forbidden optical excitations, i.e., dark states [66,67,68]. To a very good approximation, the bleach of the excitonic transition signals in these nanocrystals can be treated as solely caused by state filling of conduction band states by electrons [66,67,69,70]. The excited state absorption in nanocrystals originates from the absorption of probe light by the excited nanocrystals, leading to the formation of another exciton (i.e., biexciton). The presence of exciton modifies the energy levels (Stark effect) of the nanocrystals; therefore, the energy required to generate a second exciton is slightly different from that for the first exciton [67]. Absorption of the probe light adds positive contributions to the transient spectra. This leads to the overlap of slightly shifted bleach and the photo-absorbed signal, leading to the formation of derivative-like feature which can be easily distinguished in the spectrum. Further, intraband transitions are, in principle, possible, adding contributions in the NIR spectral range. The trapping of a hole on the surface of nanocrystals results typically in a broad photoinduced absorption band in the near-infrared region [71,72]. Stimulated emission can occur when the excited state species radiatively relaxes to the ground state, stimulated by the interaction with the probe pulse and resulting in a negative signal contribution to the overall transient spectrum. By studying the temporal development of these various signal contributions to the transient absorption spectra of nanocrystals, information about electronic dynamics occurring at ultrafast timescales, i.e., charge carrier dynamics such as relaxation to band-edge states, trapping, charge separation, electron-hole recombination, and Auger recombination, can be obtained. Furthermore, TAS can be used to study the band-alignment in nanoheterostructures which is important in designing photocatalytic systems. Hence, transient absorption spectroscopy is a highly suited method to study the mechanistic factors underlying the observed photocatalytic performance of semiconductor nanocrystal photocatalysts.

## 2. Probing Electronic Structure of Heterostructures

**Figure 2:**
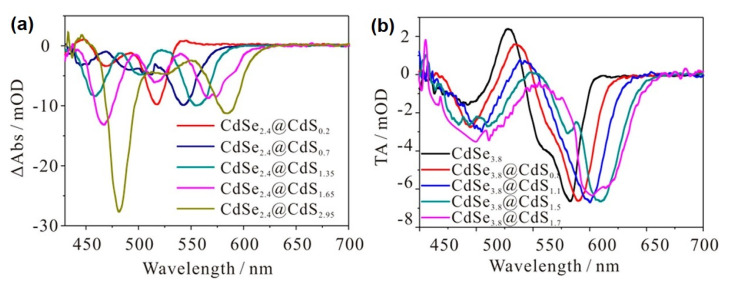
Averaged transient absorption spectra of CdSe@CdS core/shell QDs measured at 1–2 ps after excitation with lowest energy exciton excitation. (**a**) 2.4 nm and (**b**) 3.8 nm CdSe seed with increasing shell thickness. Black trace in (**b**) shows the TA spectra of the corresponding CdSe3.8 seed for comparison. Reprinted with permission from [58]. Copyright 2018 American Chemical Society.

As mentioned in the previous section, band alignment in a core/shell heterostructure influences the localization and interaction of electrons and holes in the system and significantly impacts the functionality of the system. Depending on the band-gap and relative position of electronic energy levels of the combined materials, core/shell particles can have type I, type II, and inverse type I band alignment [73]. Structures with type I band alignment have eclipsed, and those with type II band alignment have staggered electronic energy levels. Consequently, the type I system confines the electron and hole to the core material, thereby increasing the wavefunction overlap and leading to high rates for radiative recombination, whereas the type II systems support charge separation between the different domains of the heterostructure, extending the radiative lifetimes and reducing the non-radiative lifetimes due to reduced wavefunction overlap [73]. In between these two situations, in quasi-type II systems, one of the energy levels of the core is close to that of the shell material, and the respective charge carrier is delocalized over the entire system [59,74]. Therefore, it is important to know the relative positions of bands in the photocatalytic system. The band alignment at the interface of CdSe and CdS nanoparticles has been studied with different experimental methods [75,76,77,78,79] as well as theoretical methods [78,80]. Indications about band alignment can also be derived from transient absorption spectra, e.g., for CdSe@CdS particles with type-I band alignment upon selective excitation of the core material, only CdSe-related excitonic transition bleach is expected. In contrast, for quasi-type II band alignment, selective excitation of the core should also result in an immediate bleach of CdS-related excitonic transition, as the electron in quasi-type II band alignment is delocalized over the entire nanorod. For instance, Kong et al. studied the state selective excitation for establishing band level alignment by preparing a series of CdSe@CdS core/shell quantum dots with seed sizes of 2.4 and 3.8 nm (see Figure 2a,b) [58]. Upon selective excitation of the seed, smaller-seeded quantum dots showed instantaneous bleach in the shell spectral region, while larger-seeded quantum dots showed almost no bleach contribution from the shell region, confirming the quasi-type II and type I band alignment, respectively. Similar results have been shown by the near core excitation of dot-in-rod systems having seed diameters of 2.7 nm and 3.8 nm with varying length [81]. The large-seeded nanorods do not show any bleach in the 450–500 nm region, which is attributed to the CdS rod part. The small-seeded nanorods show an instantaneous bleach at the CdS rod spectral position, which is distinct from the seed-only counterpart. This observation suggests that, in the case of a larger seed size, the band alignment at the interface is type I, whereas quasi-type II band alignment is observed in the case of a smaller seed [81]. Wang et al. used state selective excitation of 2.9 nm CdSe core diameter quantum dots capped with a monolayer and bilayer CdS shell [82]. They observed that the near core excitation of core/shell quantum dots does not result in bleach in the CdS shell region, thereby inferring a type I band alignment in these systems with very thin shells [83]. This shows that even systems which are usually assigned according to the core size to be quasi-type II can show type I-like electronic situations, which was also recently observed for CdSe@CdS nanorods [82].

## 3. Charge Carrier Dynamics in Nanorods

**Figure 3:**
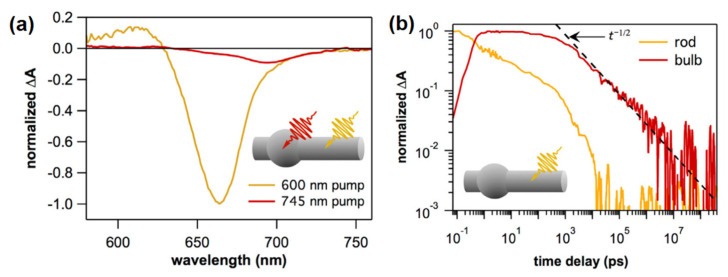
(**a**) TA spectra of CdSe rod recorded 1 ns after excitation with 600 nm (rod excitation, dark yellow color) and 745 nm (bulb excitation, red color) pump pulse. TA spectra have been normalized to have same amplitude at 705 nm. (**b**) TA kinetics of rod (dark yellow color) and bulb (red color) regions are shown up to 400 µs time window after excitation with 600 nm pump pulse and normalized to have a maximum amplitude of 1, plotted on a log–log scale. The dotted line shows the power law decay over five orders of magnitude in time. Reprinted with permission from [87]. Copyright 2018 American Chemical Society.

By analyzing the temporal development of transient spectra, transient absorption spectroscopy can reveal information about intrinsic relaxation in nanocrystals such as relaxation to band-edge states, and trapping or localization of charge carriers in subdomains of the structures. For instance, the seed-mediated synthesis of 1D CdS nanorods can induce non-uniformity in the nanorod radius, forming a bulge out region around the seed called the bulb region, which has a larger diameter than the rest of the nanorod, and consequently having lower first excitonic energy [84,85,86]. The rod and the bulb region in non-uniform CdSe (or CdS) nanorods show overlapping but distinct transient absorption features (see Figure 3a), and these features could be used to study electron-hole dynamics in transient absorption measurements. Utterback et al. observed the usual exponential decay dynamics in the rod region by directly exciting the rod, whereas a completely different decay dynamics occurs in the bulb region (see Figure 3b) [84,87,88]. The bulb region shows power-law decay, which is absent after direct excitation of the bulb region in CdSe nanorod. Also, similar power law behavior is observed in non-uniform CdS nanorods, although it is absent in the spherical CdS quantum dots and after selective excitation of the bulb region, which suggests that kinetic decay differences have their origin in the different geometries of nanorods [84]. The power-law decay is a signature of distributed kinetics (also often observed in blinking kinetics of single quantum dots) [89] such as the diffusion-limited recombination of trapped holes and stationary electrons localized in the bulb region or diffusion-annihilation recombination. Hence, this observation indicates that the hole trapping on the nanorod surface and hopping between surface trap states determine the charge carrier migration to the bulb region and, hence, the recombination dynamics [84,87]. Furthermore, the charge carrier dynamics in nanorods are largely independent of the seed material and, rather, depend on the hole trapping rate and the length of the nanorod [81,90].

**Figure 4:**
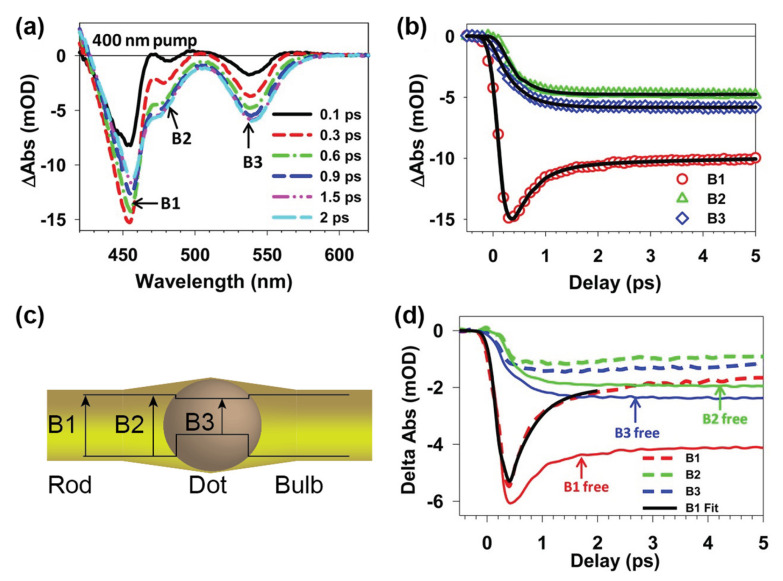
Transient absorption spectra (**a**) and TA kinetics (**b**) of CdSe@CdS nanorods after excitation with 400 nm pump pulse at indicated delay times. Reprinted with permission from [93]. Copyright 2013 American Chemical Society. (**c**) Schematic representation of B1, B2, and B3 bands. (**d**) Comparison of TA kinetics of non-tipped (solid lines) and Pt-tipped (dashed lines) rods; the black line is the fit to B1 bleach kinetics. Reprinted with permission from [55]. Copyright 2014 American Chemical Society.

Similar effects can be observed for heterostructures, i.e., nanocrystals combining regions of different materials, e.g., CdSe@CdS nanorods. In these dot-in-rod structures, besides the different domains represented by the CdSe seed and the CdS rod-shaped shell, a bulb region with higher diameter can form around the seed (see Figure 4c) [91,92]. Nanorods with such a non-uniform diameter show remarkable differences compared to those with a uniform diameter when studied carefully using single-particle spectroscopy [92]. The three distinct regions formed in DIR, namely the core, bulb, and rod region, as well as the localization of charge carriers in such complex structures, can be studied using TA spectroscopy. Wu et al., showed the formation of three distinct excitons driven by hole localization in different regions after excitation at 400 nm of CdSe@CdS nanorods, independent of band alignment. These three distinct excitons in rod, bulb, and seed regions are rather long-lived and have a half-life of 22.5, 32.1, and 8.34 ns, respectively (see Figure 4a,b) [93]. Photoluminescence spectroscopy indicates that exciton localization to the core occurs and is strongly dependent on the length of the nanorods. The longer the nanorods, the higher the probability is for the exciton to be trapped in surface trap states. This reduces the efficiency of exciton localization in the seed [81]. Using TAS, it is shown that excitons generated in the rod region relax on the timescale of 1 ps to form excitons in the energetically favored bulb and seed region, which is indicated by the increasing bleach intensity of the excitonic transitions assigned to these regions [93]. This hole localization-driven process (exciton diffusion constant was estimated to be 2.3 × 10^−4^ m^2^/s) is in competition with hole trapping, τ_Trap_ = 0.78 ± 0.13 ps, depending on the density of the trap states which are available [71,81]. Consequently, the exciton localization to the core is strongly influenced by the nature of the surface ligands and surface coverage, which influences the type and density of the available trap states. This can be used to optimize hole localization in the seed and improve the hydrogen evolution performance in tipped CdSe@CdS nanorods, due to the improved efficiency of the formation of long-lived charge separation with holes localized in the seed and electrons transferred to the metal tip (see below) [94].

## 4. Charge Transfer Dynamics in Nanorods

Transient absorption spectroscopy has been extensively used to study the charge carrier transfer dynamics to acceptor units, e.g., metal particles or molecular acceptors. Typically, the band-edge bleach recovery, reporting on the decay of the population of conduction band states, is observed to investigate the electron transfer from conduction band states to an electron acceptor. In nanocrystals in the presence of suited electron acceptors, bleach recovery occurs faster compared to the absence of acceptor units. For instance, electrons can be transferred from CdS nanorods to a metallic tip, e.g., Pt with a half-life of ~3.4 ps forming a charge separated state with half-life of ~2 µs [71,95]. By observing the bleach recovery for different excitonic transitions in non-homogeneously formed systems, forming different types of excitonic states as described above (exciton in rod, bulb, and seed regions), the rates for charge transfer from different regions of a nanostructure can be derived. CdS nanorods tipped with Pt show faster electron transfer from the rod than the bulb region or the seed, which is usually localized near the opposite end of the nanorods to the metal tip due to the increasing distance [55,71,86], e.g., CdSe/CdS DIR tipped with platinum metal tip showed electron transfers with lifetimes of nearly 1.75, 30.1, and 43.5 ps from the rod, bulb, and seed regions [55]. In this way, further relations of charge transfer rates and efficiencies on structural parameters have been derived: The charge transfer to the metal center can be affected by the composition and size of the tip, even though changes in the transfer rates can not necessarily be observed directly, but as changes in transfer efficiencies [57,96,97,98]. These directly translate into the observed hydrogen production efficiency of the photocatalysts. Also, electron transfer to molecular species can be equally fast [99,100,101]. Electron transfer from CdSe nanorods to the surface-adsorbed methyl viologen competes with the electron cooling process and both processes occur in a range from approximately 0.5 ps to a few picoseconds. The ZnS shell on CdSe nanorods forming type I band alignment slows down the electron transfer by introducing an energy barrier. CdSe nanorods coated with a ZnS shell of 1 nm thickness showed 25–50 times slower electron transfer compared to bare CdSe nanorods [102].

Often, the hole transfer to the quencher is the rate limiting step in the photocatalytic process [55]. Therefore, it is necessary to understand and remove the bottlenecks of the hole transfer process. The kinetics of hole trapping can be observed directly via the broad photoinduced absorption band below the band-edge in these semiconductor nanocrystals, which is indicative of trapped holes. This feature builds up within a timescale <1 ps [71,81]. Vice versa, processes which quench the trapped holes can be observed via the decay of this feature [103,104]. However, as mentioned above, hole transfer dynamics from valence band states in II-VI nanocrystals cannot be observed directly in transient absorption spectroscopy, due to a lack of contributions to the TA signal caused by the high spectral density of valence band states [66]. A set of control experiments and, indirectly, the delayed onset of the CdS nanorod band-edge decay (representing electron population transfer) in the presence of Ru-based water oxidation catalyst acting as a hole acceptor, was used to estimate the timescale of hole transfer to occur from the 100 ps to 1 ns timescale [105]. Alternatively, hole transfer can be observed through the formation of a spectral signature of the oxidized acceptor, which can be detected in the transient spectra [30,106]. For example, phenothiazine attached to CdS nanorods can act as a hole acceptor and the kinetics of formation of oxidized phenothiazine can be observed at ~520 nm to probe the kinetics of trapped holes. The trapped holes from CdS can be transferred with a half-life of 3.8 ns, generating a charge separated state with a half-life of 310 ns and with an extraction efficiency of 95% [30]. The hole transfer rate is strongly dependent on the number of acceptors, as has been shown for the transfer from quantum dots to phenothiazine [106,107]. For example, CdSe-phenothiazine shows hole transfer ranging from 2.5 ns in a 1:1 molar ratio to 300 ps in a 1:6 CdSe:phenothiazine molar ratio [106]. If direct spectroscopic features of a ligand are absent, transient absorption spectroscopy can only be used in combination with other techniques, e.g., photoluminescence upconversion, to provide insights into the valence band hole transfer dynamics [108].

**Figure 5:**
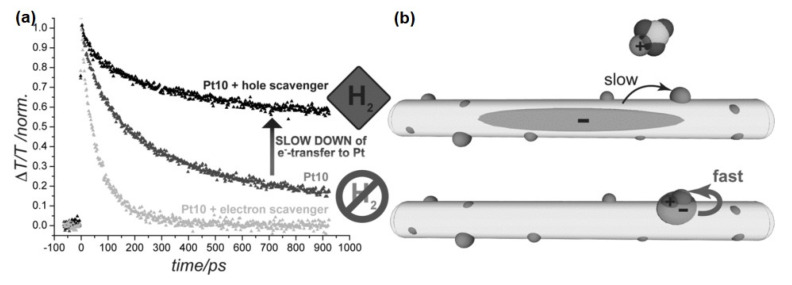
(**a**) TA kinetics of Pt-decorated CdS (Pt10) nanorods excited with 400 nm pump pulse at CdS band-edge (dark grey), in the presence of methyl viologen as electron scavenger (grey trace) and in the presence of sodium sulfite as hole scavenger (dark black). (**b**) Schematic of underlying physical process in presence (upper) and absence (lower) of hole scavenger. Reprinted with permission from [109]. Copyright 2012 WILEY-VCH Verlag GmbH & Co. KGaA, Weinheim.

The charge transfer investigations mentioned above were performed in the absence of a hole (or electron) quencher. The quenching of the opposite charge carrier on similar timescales could impact the charge transfer step. For CdS nanorods decorated with a large number of Pt metal particles, Berr et al. observed that bleach recovery is slowed down in the presence of a hole scavenger (see Figure 5a) [109]. This observation is rationalized by the reduction in spatial overlap between the electron wavefunction to metallic particles due to the enhanced delocalization in the absence of trapped holes (see schematic Figure 5b) [109]. It is to be noted that, in this system, Pt particles are distributed over the whole surface of the nanorods. By trapping holes in surface trap sites, electrons also localized close to this site, which has a high probability of being close to a Pt acceptor particle. This example nicely illustrates that, to fully understand the dynamics in an active system, experiments in the presence of sacrificial agents are necessary, though only performed in rare cases.

## 5. Multiple Charge Carrier Accumulation and Transfer

The studies discussed above only focus on the transfer of a single electron to the reaction center. However, multielectron transfer and accumulation are required to drive the multielectron redox reactions involved in solar-to-fuel conversion. For instance, hydrogen generation from water requires two electrons. Therefore, several photon absorption and transfer events are required to accumulate the required number of electrons for the target reaction at the active site. In this respect, several questions arise: How does the transfer of several charges occur most efficiently? How does the presence of sacrificial agents or the number of acceptors influence the transfer of multiple charge carriers? How can charge carrier recombination be prevented? Can multiple exciton formation and quasi-simultaneous transfer be exploited to improve activity compared to consecutive charge transfer processes? There are only a limited number of studies addressing these challenges directly, although, as was described above, to develop a detailed understanding of the single mechanistic steps in time resolved spectroscopic studies, the multielectron nature of the catalytic process must be taken into the equation.

**Figure 6:**
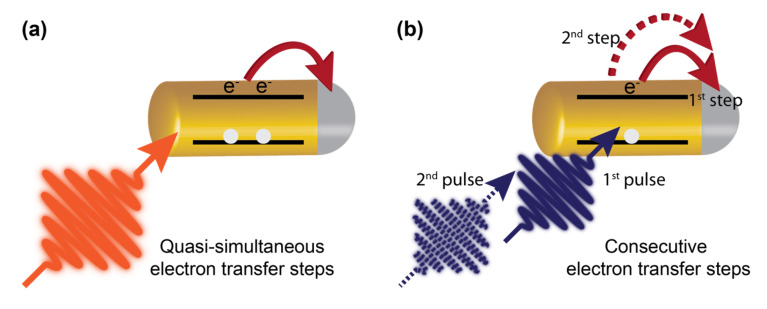
Schematic representation of two limiting cases for multi-electron transfer: (**a**) Quasi-simultaneous electron transfer and (**b**) consecutive electron transfer process.

The multielectron photocatalytic reduction might follow a cascade type mechanism, i.e., the consecutive absorption of photons and sequential transfer of electrons (see schematic Figure 6b) or the quasi-simultaneous absorption of photons and the formation of multiple excitons and the transfer of electrons to the cocatalyst (see schematic Figure 6a). In a consecutive electron transfer type mechanism, the dissociation of the exciton and the transfer of the first electron to the cocatalyst leaves the hole behind; hence, the subsequent photoabsorption could form a charged exciton rather than a neutral exciton, unless quenching of the remaining hole occurs before the second excitation via interaction with the sacrificial electron donor. How the potential accumulation of holes impacts the following charge transfer steps or whether the charging of the acceptor affects the following charge transfer steps is a question to be studied. In contrast, in the case of simultaneous electron transfer, the electron transfer step must compete with the rather efficient Auger recombination process in nanocrystals, an interplay to be studied to gain control over these processes and to exploit multiple exciton effects in light harvesting.

**Figure 7:**
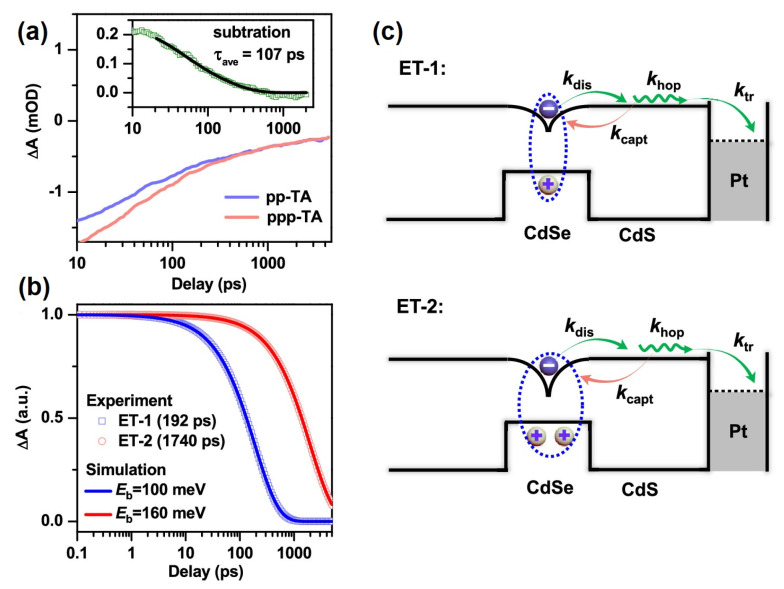
(**a**) TA bleach recovery kinetics of CdSe@CdS dot-in-rod monitored at 560 nm (CdSe rod) after excitation with 520 nm pump in a pump–probe (pp; blue line) and pump–pump–probe (ppp; red line) experiment. The kinetics have been scaled to the long-lived component. Inset: Subtracted kinetics to obtain pure ppp-TA kinetics (green squares) and its biexponential fit (black line) giving average lifetime of 107 ps. (**b**) Extracted first electron ET-1 (blue squares) and second ET-2 (red circles) transfer kinetics. The solid lines are the fits. (**c**) Schematic diagram to show dissociation-limited long-range electron transfer from the dot to the Pt-metal tip from neutral (upper) and positively charged (lower) excitons. *k*_dis_, *k*_capt_, *k*_hop_, and *k*_tr_ stand for the rates of electron dissociation, recapturing by the dot, hopping along the rod, and trapped to the tip, respectively. Reproduced with permission from [117]. Copyright © 2020, American Chemical Society.

To study the cascading nature of electron transfer using transient absorption is challenging because the intermediate species generated by pump pulse excitation are rather short lived and can be prepared only in low concentrations. To generate the intermediate species, (electro)chemical doping methods can give access to intermediates in electron transfer cascades if the species are sufficiently long-lived. Alternatively, a pump-pump-probe (PPP) spectroscopic scheme could be applied. In PPP spectroscopy, a sequence of three pulses is used. The first pump pulse excites the systems and is used to prepare in situ an intermediate of the light-induced electron transfer cascade. The second pump pulse, together with the probe pulse, allows for investigating the dynamics in this intermediate, as in normal TA schemes. In this way, PPP spectroscopy allows for performing classical transient absorption spectroscopy on a transiently generated species. PPP spectroscopy has been used in molecular [110,111,112,113,114,115] and nanocrystalline systems [116,117] to monitor charge recombination and accumulation processes, e.g., the PPP technique was used for the photoinduced doping of nanocrystals to study the lifetime of the trion state formed upon the excitation of a charged nanocrystal [116,117,118,119]. In this study, the first pulse selectively excites the nanocrystals in a CsPbBr_3_–rhodamine B complex, triggering charge transfer. The second pulse interacts with the charged species, or the fraction of species, thereby generating a trion state instead of a neutral excitonic species. Positive and negative trion lifetimes of 220 ± 50 ps and 150 ± 40 ps are determined this way, respectively. Further, it is shown that the efficiency of electron transfer from a positive trion state reduces to 65.4 ± 4.2% compared to 89.8 ± 3.6% from a neutral NC [116]. The observed reduction in efficiency of charge transfer from a charged excitonic state is important, as it would also impact a cascade-type electron in photocatalysts in the absence of sacrificial agents. The second electron transfer step from a trion state would be required to compete with the faster Auger recombination of a trion state. Indeed, similar observations have been made for CdSe@CdS DIR nanocrystals tipped with Pt particles. Applying PPP spectroscopy, the rate for a second electron transfer step was determined (see Figure 7a,b). It was shown in the absence of a hole quencher that the second electron transfer step to the cocatalyst is much slower. The first electron transfer step in CdSe/CdS DIR systems occurs with a time constant of 192 ps and an efficiency of 97.4%, whereas the second electron transfer step occurs with a timescale of 1740 ps, which is nearly an order of magnitude higher and shows an efficiency of only 6.1% (see Figure 7b) [117]. This is explained by an increase in the barrier for the electron transfer to the metal tip by nearly 60 meV, which is caused by two holes remaining in the semiconductor nanostructure. Since the second electron transfer step must compete, additionally, with the Auger recombination of the trion, the state efficiency of the second electron transfer step is low (see schematic Figure 7c). This indicates that the presence of a sacrificial agent might not only be required to prevent photooxidation by hole quenching, but also to enable an efficient second electron transfer by removing the hole before a second excitation and charge-transfer occurs. This still must be proven.

Compared to standard transient absorption spectroscopy, the small number of PPP studies indicate the challenges this technique presents for setting certain experimental conditions and data evaluation, e.g., for studying sequential charge transfer steps by PPP transient absorption spectroscopy, one needs to account for the direct reabsorption of the second pump pulse by ground state species, which would be identical to the standard pump probe experiment. Further, the influence of higher order exciton (such as biexciton) also must be accounted for, since more than one photon can be absorbed by the nanorods. This superposition of dynamics in the collected data challenges data evaluation routines, and the development of strategies addressing these issues must be carried out before this method can be applied for such studies on a routine basis.

In consecutive charge transfer pathways, recombination before sufficient charge carriers can accumulate can be an efficiency limiting factor. To circumvent this, the simultaneous transfer of charge carriers might be an option. Multiple excitons can be formed by absorbing more than one photon by single nanocrystals [66,120,121,122] or by absorbing a high energy single photon which then dissociates to form multiple excitons in a process known as carrier multiplication (CM) [123,124,125]. The dissociation of multiple excitons and the transfer of multiple charge carriers to the catalytic reaction center could support accumulating the required charge carriers at the catalytic site. This process has been proposed to be responsible for the relative increase in photocatalytic activity under high intensity excitation conditions [126]. To be able to exploit this effect, the properties of multiple excitons must be studied. Most studies apply TAS in the multipole excitonic regime, using pump intensities that are high enough to generate multiple excitons per nanocrystal. The band-edge bleach decay kinetics under high intensity excitation conditions shows an additional ultrafast decay feature compared to the decay kinetics in the single-exciton regime on the tens to hundreds of picosecond timescale. The ultrafast time component can be attributed to the fast depletion of high-order excitons governed by many-body interactions, i.e., Auger recombination to monoexcitons. The rate of the Auger recombination process is dependent on the initial order (N) of the exciton that is generated. Statistically, for strongly confined systems such as quantum dots, the relation $k_{AR}\propto N^{2}\left(N-1\right),$ and for loosely confined systems such as nanorods $k_{AR}\propto N\left(N-1\right),$ are expected to describe the Auger recombination rate $k_{AR}$ [66,122]. Wave-function engineering, reducing the electron-hole overlap and coulombic interactions, can lead to prolonged biexcitonic lifetimes. Further, 2.4 nm core-sized CdSe@CdS core/shell QDs of shell thickness ~3 nm showed a 40-fold increase in biexciton lifetime, while 3.8 nm core-sized QDs of shell thickness ~1 nm only showed a 4-fold increase in biexciton lifetimes when compared to those of bare core quantum dots [58]. Similar results have also been reported in CdSe seeds embedded in a one-dimensional elongated CdS shell, i.e., dot-in-rod nanostructures [81,93,127].

**Figure 8:**
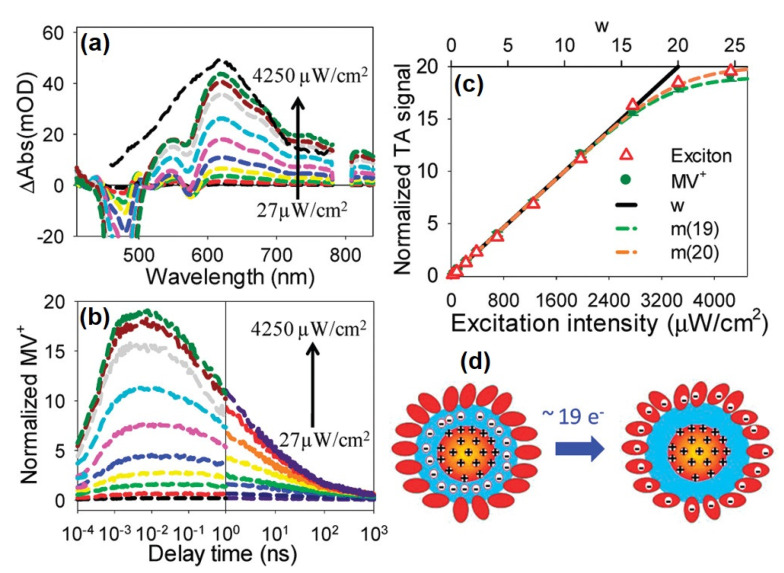
(**a**) Averaged transient absorption spectra of CdSe/CdS–MV^2+^ core/shell quantum dot at 8–10 ps at indicated pump intensities. (**b**) Normalized kinetic traces of MV^+^ radical (630 nm) with increasing pump intensity. (**c**) Normalized MV^+^ radical signal (at 8–10 ps) in QD–MV^2+^ complexes as a function of excitation intensities. (**d**) Schematic representation of ultrafast transfer of 19 e^−^ from core/shell quantum dot to the adsorbed MV^2+^ molecules. Reproduced with permission from [128]. Copyright © 2012, American Chemical Society.

Because Auger recombination occurs on a few to hundreds of picoseconds timescale, to extract multiple charge carriers from multiple excitons, the multiple exciton dissociation to transfer charge carriers must occur on a similar or even shorter timescale to compete efficiently with the Auger recombination process. CdSe and CdSe/CdS quantum dots have been shown to transfer multiple electrons to electron acceptors such as methyl viologen [101,128] and methylene blue [99,122] on an ultrafast timescale, resulting in a long-lived charge-separated state. For example, at high pump intensity, the transfer of up to 19 e^−^ has been demonstrated to occur efficiently from quasi-type II CdSe/CdS core/shell quantum dots (see Figure 8). The transient absorption spectra show the formation of reduced methyl viologen (MV^+^) at 630 nm (Figure 8a). The intensity of the spectral band at 630 nm increases with pump fluence and saturates at high fluence (see Figure 8a,b), corresponding to the formation of more reduced MV^+^ species at high fluence. The number of reduced MV^+^ can be estimated by looking at the normalized transient absorption signal as a function of pump fluence (see Figure 8c,d) under the assumption that, at low intensity, all of the excitons generated in quantum dots have transferred an electron to methyl viologen ligand, thus allowing for estimation of the number of reduced MV^+^ radicals per quantum dot under high pump fluence. This transfer of multiple excitons occurs within 0.18 ps, which is fast compared to the biexcitonic lifetime of 440 ps [128]. Likewise, multiple photogenerated holes have also been shown to be transferred to molecular hole acceptor ligands. Yan et al. showed that the hole transfer rate from CdS quantum dots could compete with the Auger recombination process [129]. Multiple holes (~three holes when excited with <N_0_> = 19) can be transferred to 4-(3-bromo-7-(dihexylamino)-10H-phenothiazin-10yl)benzoic acid (NPTZ) attached to CdS quantum dots. In contrast, CdSe and CdSe/CdS quantum dots used in the same study clearly showed that the Auger recombination rate outcompetes the hole transfer process for these systems, and only a few holes which were transferred to the NPTZ ligand [129]. Furthermore, the shape of the nanocrystal can be modified to further increase the number of electrons transferred since the rate of Auger recombination decay depends on the shape of the system [130]. Under high excitation power, CdSe nanorods with a biexciton lifetime of 201 ps show 21 electron transfers, whereas CdSe quantum dots with a biexciton lifetime of 50 ps only show 4 electron transfers to the absorbed methyl viologen molecules [130]. However, it is important to note that the transfer of multiple electrons/holes does not necessarily lead to the accumulation of charge carriers at an active site to drive a chemical reaction, as stated earlier. The presence of multiple acceptors might lead to a decrease in catalytic efficiency [41].

**Figure 9:**
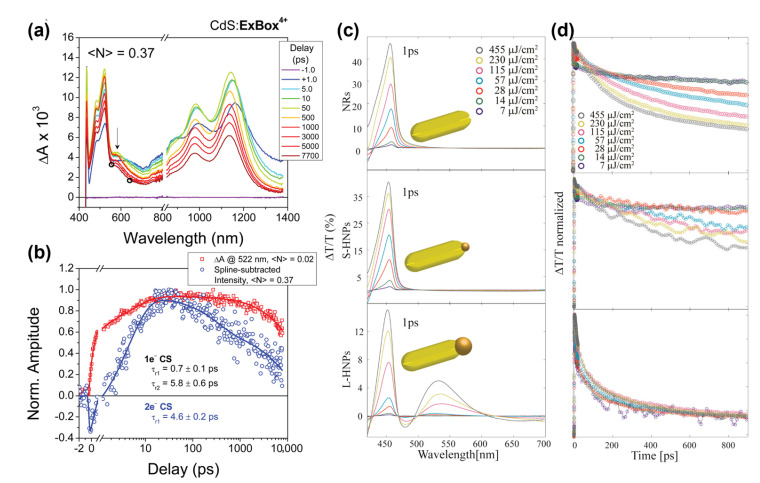
(**a**) Transient absorption spectra of CdS:ExBox^4+^ excited with 414 nm pump with <N> = 0.37 (1.0 mW). At this higher excitation intensity, a new band emerges at 588 nm (marked with an arrow), characteristic of ExBox^2(+•)^. Black circles indicate the wavelengths for spline-subtraction needed to extract the kinetics of second electron transfer process. (**b**) Comparison of the normalized spline-subtracted kinetics of CdS^2(+•)^:ExBox^2(+•)^ at <*N*> = 0.37 (blue) with CdS^+•^:ExBox^3+•^ obtained at <*N*> = 0.02 (red). Reproduced with permission from [131]. Copyright © 2016, American Chemical Society. (**c**) Differential transmittance spectra of CdS NRs and CdS–Au hybrid nanoparticles having different Au metal tip sizes of 1.5 nm (S-HNPs) and 7.1 nm (L-HNPs) excited with 400 nm pump pulse with varying fluences after 1 ps of excitation. (**d**) Corresponding band-edge bleach recovery kinetics of CdS and CdS–Au NR normalized to maximum bleach point. Reproduced with permission from [126]. Copyright © 2018, American Chemical Society.

Also, Young et al. have shown the transfer of two electrons from CdS quantum dots in a 1:1 mixture with extended viologen cyclophane when excited with a sufficiently high intensity pump pulse [131]. Figure 9a shows the transient absorption spectra (excitation density <N> = 0.37) of CdS quantum dots with tetracationic compound cyclobis(4,4′-(1,4-phenylene) bipyridin-1-ium-1,4-phenylene-bis(methylene)) (ExBox^4+^) adsorbed to its surface. The transient absorption spectra show an additional band at 588 nm under high fluence conditions corresponding to ExBox^2(+•)^. The first and second electron transfer kinetics are monitored by following the decay kinetics of the 522 nm and 588 nm bands (as shown by the steady state absorption of chemically reduced ExBox) under low fluence and high pump fluence, respectively (see Figure 9b). Both the electron transfer steps occur at the ultrafast timescale of 1 and 5 ps, respectively. The doubly reduced complex has a lifetime of ~10 ns [131]. Metallic clusters as an electron sink might be a better choice than molecular acceptors, as Liu et al. showed a 100% multiple exciton dissociation and transfer from CdS nanorods to the Pt tip from a biexcitonic state, decreasing to ~41% at <N> ~ 23 [132]. Also, the Banin group showed that Au metal tips of a suitable diameter can directly accept multiple electrons by outcompeting the Auger recombination process in the CdS nanorods [126]. The intensity dependent differential transmittance spectra of non-tipped CdS, and small and large metal-tipped CdS-Au NRs are shown in Figure 9c. The kinetic trace of the CdS band edge bleach shows an additional kinetic component with increasing the intensity of the pump beam characteristic of contributions from Auger recombination at high intensities in the non-tipped system (see Figure 9d), whereas the decay kinetics of small and large metal-tipped CdS NR is essentially independent of the pump intensity, showing the transfer of photogenerated multiple electrons to the metal tip. The large-tipped samples showed increased relative photocatalytic activity compared to the small-tipped samples in the higher excitonic regime [126].

However, there are challenges associated with these mechanisms which must be overcome to critically assess their role in driving photocatalytical reactions. For the simultaneous electron type transfer mechanism, their commercial applicability might be limited since under one-sun illumination, it is rather hard to access the nonlinear excitation conditions without using, e.g., solar concentrators or plasmonic particles to enhance the absorption rate in the near field enhancement limit. Therefore, the multielectron photocatalytic reduction might follow a cascade type mechanism, i.e., the consecutive absorption of photons and sequential transfer of electrons to the cocatalyst. Even under high pump fluences, CdS quantum dots mixed with extended cyclophane hints towards the sequential electron transfer steps rather than a simultaneous transfer step [131]. Up until now, too few exemplary systems have been studied in a multiple exciton regime to assess their influence in photocatalysis.

## 6. Conclusions and Outlook

The solar-to-fuel conversion application of semiconductor nanocrystals holds immense potential due to their high visible light absorption properties and their easy charge separation and migration to the catalytic site, making them suitable candidates for artificial photocatalysis. In this review, we present a comprehensive overview on the investigation of charge carrier dynamics in semiconductor nanocrystals in the context of application in light-driven catalysis. The special role of transient absorption spectroscopy as the method of choice is highlighted here. The application of this technique supports the growing understanding of mechanisms underlying the photocatalytic activity and the influence of various design parameters such as shape, band-gap engineering, nature and number of co-catalytic sites on the charge separation, and migration properties of semiconductor nanocrystals. A particular challenge for researchers is the need to transfer multiple charge carriers, which is usually not explicitly addressed in most investigations. In this review, we summarize the few existing investigations which take up these challenges, especially those addressing multiple charge carrier events and considering potential consecutive charge transfer processes or processes involving multiple excitons. Interest in these effects is growing and we expect growing activity addressing these effects in the coming years.

## Data Availability

Not applicable.

## References

[1] Lesnyak V., Gaponik N., Eychmüller A. (2013). Colloidal Semiconductor Nanocrystals: The Aqueous Approach. Chem. Soc. Rev..

[2] Chaudhuri R.G., Paria S. (2012). Core/Shell Nanoparticles: Classes, Properties, Synthesis Mechanisms, Characterization, and Applications. Chem. Rev..

[3] Talapin D.V., Lee J.S., Kovalenko M.V., Shevchenko E.V. (2010). Prospects of Colloidal Nanocrystals for Electronic and Optoelectronic Applications. Chem. Rev..

[4] Pietryga J.M., Park Y.-S., Lim J., Fidler A.F., Bae W.K., Brovelli S., Klimov V.I. (2016). Spectroscopic and Device Aspects of Nanocrystal Quantum Dots. Chem. Rev..

[5] Gaponik N., Hickey S.G., Dorfs D., Rogach A.L., Eychmüller A. (2010). Progress in the Light Emission of Colloidal Semiconductor Nanocrystals. Small.

[6] Vanmaekelbergh D., Liljeroth P. (2005). Electron-Conducting Quantum Dot Solids: Novel Materials Based on Colloidal Semiconductor Nanocrystals. Chem. Soc. Rev..

[7] Li X.-B., Tung C.-H., Wu L.-Z. (2018). Semiconducting Quantum Dots for Artificial Photosynthesis. Nat. Rev. Chem..

[8] Stolarczyk J.K., Bhattacharyya S., Polavarapu L., Feldmann J. (2018). Challenges and Prospects in Solar Water Splitting and CO_2_ Reduction with Inorganic and Hybrid Nanostructures. ACS Catal..

[9] Maeda K., Domen K. (2010). Photocatalytic Water Splitting: Recent Progress and Future Challenges. J. Phys. Chem. Lett..

[10] Izadpanah Ostad M., Niknam Shahrak M., Galli F. (2021). Photocatalytic Carbon Dioxide Reduction to Methanol Catalyzed by ZnO, Pt, Au, and Cu Nanoparticles Decorated Zeolitic Imidazolate Framework-8. J. CO2 Util..

[11] Kumar S., Regue M., Isaacs M.A., Freeman E., Eslava S. (2020). All-Inorganic CsPbBr _3_ Nanocrystals: Gram-Scale Mechanochemical Synthesis and Selective Photocatalytic CO_2_ Reduction to Methane. ACS Appl. Energy Mater..

[12] Choi J.Y., Lim C.K., Park B., Kim M., Jamal A., Song H. (2019). Surface Activation of Cobalt Oxide Nanoparticles for Photocatalytic Carbon Dioxide Reduction to Methane. J. Mater. Chem. A.

[13] Feng Y.-X., Wang H.-J., Wang J.-W., Zhang W., Zhang M., Lu T.-B. (2021). Stand-Alone CdS Nanocrystals for Photocatalytic CO_2_ Reduction with High Efficiency and Selectivity. ACS Appl. Mater. Interfaces.

[14] Maeda K., Domen K. (2007). New Non-Oxide Photocatalysts Designed for Overall Water Splitting under Visible Light. J. Phys. Chem. C.

[15] Moroz P., Boddy A., Zamkov M. (2018). Challenges and Prospects of Photocatalytic Applications Utilizing Semiconductor Nanocrystals. Front. Chem..

[16] Lin Y., Yuan G., Liu R., Zhou S., Sheehan S.W., Wang D. (2011). Semiconductor Nanostructure-Based Photoelectrochemical Water Splitting: A Brief Review. Chem. Phys. Lett..

[17] Vaneski A., Schneider J., Susha A.S., Rogach A.L. (2014). Colloidal Hybrid Heterostructures Based on II-VI Semiconductor Nanocrystals for Photocatalytic Hydrogen Generation. J. Photochem. Photobiol. C Photochem. Rev..

[18] Wu K., Lian T. (2016). Quantum Confined Colloidal Nanorod Heterostructures for Solar-to-Fuel Conversion. Chem. Soc. Rev..

[19] Zhang B., Sun L. (2019). Artificial Photosynthesis: Opportunities and Challenges of Molecular Catalysts. Chem. Soc. Rev..

[20] Li X., Yu J., Low J., Fang Y., Xiao J., Chen X. (2015). Engineering Heterogeneous Semiconductors for Solar Water Splitting. J. Mater. Chem. A.

[21] Maeda K. (2013). Z-Scheme Water Splitting Using Two Different Semiconductor Photocatalysts. ACS Catal..

[22] Wilker M.B., Schnitzenbaumer K.J., Dukovic G. (2012). Recent Progress in Photocatalysis Mediated by Colloidal II-VI Nanocrystals. Isr. J. Chem..

[23] Peng X., Schlamp M.C., Kadavanich A.V., Alivisatos A.P. (1997). Epitaxial Growth of Highly Luminescent CdSe/CdS Core/Shell Nanocrystals with Photostability and Electronic Accessibility. J. Am. Chem. Soc..

[24] Talapin D.V., Koeppe R., Götzinger S., Kornowski A., Lupton J.M., Rogach A.L., Benson O., Feldmann J., Weller H. (2003). Highly Emissive Colloidal CdSe/CdS Heterostructures of Mixed Dimensionality. Nano Lett..

[25] Yang J., Wang D., Han H., Li C. (2013). Roles of Cocatalysts in Photocatalysis and Photoelectrocatalysis. Acc. Chem. Res..

[26] Xu Y., Xu R. (2015). Nickel-Based Cocatalysts for Photocatalytic Hydrogen Production. Appl. Surf. Sci..

[27] Ran J., Zhang J., Yu J., Jaroniec M., Qiao S.Z. (2014). Earth-Abundant Cocatalysts for Semiconductor-Based Photocatalytic Water Splitting. Chem. Soc. Rev..

[28] Han B., Hu Y.H. (2016). MoS_2_ as a Co-catalyst for Photocatalytic Hydrogen Production from Water. Energy Sci. Eng..

[29] Chen X., Shen S., Guo L., Mao S.S. (2010). Semiconductor-Based Photocatalytic Hydrogen Generation. Chem. Rev..

[30] Wu K., Du Y., Tang H., Chen Z., Lian T. (2015). Efficient Extraction of Trapped Holes from Colloidal CdS Nanorods. J. Am. Chem. Soc..

[31] Wolff C.M., Frischmann P.D., Schulze M., Bohn B.J., Wein R., Livadas P., Carlson M.T., Jäckel F., Feldmann J., Würthner F. (2018). All-in-One Visible-Light-Driven Water Splitting by Combining Nanoparticulate and Molecular Co-Catalysts on CdS Nanorods. Nat. Energy.

[32] Spanhel L., Haase M., Weller H., Henglein A. (1987). Photochemistry of Colloidal Semiconductors. 20. Surface Modification and Stability of Strong Luminescing CdS Particles. J. Am. Chem. Soc..

[33] Chen H., Gai H., Yeung E.S. (2009). Inhibition of Photobleaching and Blue Shift in Quantum Dots. Chem. Commun..

[34] Pietra F., van Dijk-Moes R.J.A., Ke X., Bals S., Van Tendeloo G., de Mello Donega C., Vanmaekelbergh D. (2013). Synthesis of Highly Luminescent Silica-Coated CdSe/CdS Nanorods. Chem. Mater..

[35] Reiss P., Bleuse J., Pron A. (2002). Highly Luminescent CdSe/ZnSe Core/Shell Nanocrystals of Low Size Dispersion. Nano Lett..

[36] Wen X., Sitt A., Yu P., Ko H., Toh Y.-R., Tang J. (2012). Studies of the Photostability of CdSe/CdS Dot-in-Rod Nanoparticles. J. Nanoparticle Res..

[37] Amirav L., Alivisatos A.P. (2010). Photocatalytic Hydrogen Production with Tunable Nanorod Heterostructures. J. Phys. Chem. Lett..

[38] Perry D., Waiskopf N., Verbitsky L., Remennik S., Banin U. (2019). Shell Stabilization of Photocatalytic ZnSe Nanorods. ChemCatChem.

[39] Berr M.J., Wagner P., Fischbach S., Vaneski A., Schneider J., Susha A.S., Rogach A.L., Jäckel F., Feldmann J. (2012). Hole Scavenger Redox Potentials Determine Quantum Efficiency and Stability of Pt-Decorated CdS Nanorods for Photocatalytic Hydrogen Generation. Appl. Phys. Lett..

[40] Bang J.U., Lee S.J., Jang J.S., Choi W., Song H. (2012). Geometric Effect of Single or Double Metal-Tipped CdSe Nanorods on Photocatalytic H2 Generation. J. Phys. Chem. Lett..

[41] Nakibli Y., Kalisman P., Amirav L. (2015). Less Is More: The Case of Metal Cocatalysts. J. Phys. Chem. Lett..

[42] Choi J.Y., Park W.-W., Park B., Sul S., Kwon O.-H., Song H. (2021). Optimal Length of Hybrid Metal–Semiconductor Nanorods for Photocatalytic Hydrogen Generation. ACS Catal..

[43] Simon T., Carlson M.T., Stolarczyk J.K., Feldmann J. (2016). Electron Transfer Rate vs Recombination Losses in Photocatalytic H_2_ Generation on Pt-Decorated CdS Nanorods. ACS Energy Lett..

[44] Chen W., Li X., Wang F., Javaid S., Pang Y., Chen J., Yin Z., Wang S., Li Y., Jia G. (2020). Nonepitaxial Gold-Tipped ZnSe Hybrid Nanorods for Efficient Photocatalytic Hydrogen Production. Small.

[45] Acharya K.P., Khnayzer R.S., O’Connor T., Diederich G., Kirsanova M., Klinkova A., Roth D., Kinder E., Imboden M., Zamkov M. (2011). The Role of Hole Localization in Sacrificial Hydrogen Production by Semiconductor-Metal Heterostructured Nanocrystals. Nano Lett..

[46] Boecker M., Micheel M., Mengele A.K., Neumann C., Herberger T., Marchesi D’Alvise T., Liu B., Undisz A., Rau S., Turchanin A. (2021). Rhodium-Complex-Functionalized and Polydopamine-Coated CdSe@CdS Nanorods for Photocatalytic NAD^+^ Reduction. ACS Appl. Nano Mater..

[47] Bie C., Fu J., Cheng B., Zhang L. (2018). Ultrathin CdS Nanosheets with Tunable Thickness and Efficient Photocatalytic Hydrogen Generation. Appl. Surf. Sci..

[48] Jia J., Sun W., Zhang Q., Zhang X., Hu X., Liu E., Fan J. (2020). Inter-Plane Heterojunctions within 2D/2D FeSe_2_/g-C_3_N_4_ Nanosheet Semiconductors for Photocatalytic Hydrogen Generation. Appl. Catal. B Environ..

[49] Wu K., Zhu H., Lian T. (2015). Ultrafast Exciton Dynamics and Light-Driven H_2_ Evolution in Colloidal Semiconductor Nanorods and Pt-Tipped Nanorods. Acc. Chem. Res..

[50] Banin U., Ben-Shahar Y., Vinokurov K. (2014). Hybrid Semiconductor-Metal Nanoparticles: From Architecture to Function. Chem. Mater..

[51] Giblin J., Kuno M. (2010). Nanostructure Absorption: A Comparative Study of Nanowire and Colloidal Quantum Dot Absorption Cross Sections. J. Phys. Chem. Lett..

[52] Carey C.R., LeBel T., Crisostomo D., Giblin J., Kuno M., Hartland G.V. (2010). Imaging and Absolute Extinction Cross-Section Measurements of Nanorods and Nanowires through Polarization Modulation Microscopy. J. Phys. Chem. C.

[53] Berr M., Vaneski A., Susha A.S., Rodríguez-Fernández J., Döblinger M., Jäckel F., Rogach A.L., Feldmann J. (2010). Colloidal CdS Nanorods Decorated with Subnanometer Sized Pt Clusters for Photocatalytic Hydrogen Generation. Appl. Phys. Lett..

[54] Vaneski A., Susha A.S., Rodríguez-Fernández J., Berr M., Jäckel F., Feldmann J., Rogach A.L. (2011). Hybrid Colloidal Heterostructures of Anisotropic Semiconductor Nanocrystals Decorated with Noble Metals: Synthesis and Function. Adv. Funct. Mater..

[55] Wu K., Chen Z., Lv H., Zhu H., Hill C.L., Lian T. (2014). Hole Removal Rate Limits Photodriven H_2_ Generation Efficiency in CdS-Pt and CdSe/CdS-Pt Semiconductor Nanorod-Metal Tip Heterostructures. J. Am. Chem. Soc..

[56] Kalisman P., Nakibli Y., Amirav L. (2016). Perfect Photon-to-Hydrogen Conversion Efficiency. Nano Lett..

[57] Wächtler M., Kalisman P., Amirav L. (2016). Charge-Transfer Dynamics in Nanorod Photocatalysts with Bimetallic Metal Tips. J. Phys. Chem. C.

[58] Kong D., Jia Y., Ren Y., Xie Z., Wu K., Lian T. (2018). Shell-Thickness-Dependent Biexciton Lifetime in Type I and Quasi-Type II CdSe@CdS Core/Shell Quantum Dots. J. Phys. Chem. C.

[59] Müller J., Lupton J.M., Lagoudakis P.G., Schindler F., Koeppe R., Rogach A.L., Feldmann J., Talapin D.V., Weller H. (2005). Wave Function Engineering in Elongated Semiconductor Nanocrystals with Heterogeneous Carrier Confinement. Nano Lett..

[60] Irfan R.M., Jiang D., Sun Z., Lu D., Du P. (2016). Enhanced Photocatalytic H2 Production on CdS Nanorods with Simple Molecular Bidentate Cobalt Complexes as Cocatalysts under Visible Light. Dalton Trans..

[61] Simon T., Bouchonville N., Berr M.J., Vaneski A., Adrović A., Volbers D., Wyrwich R., Döblinger M., Susha A.S., Rogach A.L. (2014). Redox Shuttle Mechanism Enhances Photocatalytic H_2_ Generation on Ni-Decorated CdS Nanorods. Nat. Mater..

[62] Dong K., Le T., Nakibli Y., Schleusener A., Wächtler M., Amirav L. (2022). Molecular Metallocorrole–Nanorod Photocatalytic System for Sustainable Hydrogen Production. ChemSusChem.

[63] Ruhman S. (2021). Solving Quantum-Dot Excitonic Riddles with Absolute Pump–Probe Spectroscopy. J. Phys. Chem. Lett..

[64] Knowles K.E., McArthur E.A., Weiss E.A. (2011). A Multi-Timescale Map of Radiative and Nonradiative Decay Pathways for Excitons in CdSe Quantum Dots. ACS Nano.

[65] Zhang C., Do T.N., Ong X., Chan Y., Tan H.-S. (2016). Understanding the Features in the Ultrafast Transient Absorption Spectra of CdSe Quantum Dots. Chem. Phys..

[66] Klimov V.I. (2007). Spectral and Dynamical Properties of Multiexcitons in Semiconductor Nanocrystals. Annu. Rev. Phys. Chem..

[67] Klimov V.I. (2000). Optical Nonlinearities and Ultrafast Carrier Dynamics in Semiconductor Nanocrystals. J. Phys. Chem. B.

[68] Dana J., Haggag O.S., Dehnel J., Mor M., Lifshitz E., Ruhman S. (2021). Testing the Fate of Nascent Holes in CdSe Nanocrystals with Sub-10 Fs Pump–Probe Spectroscopy. Nanoscale.

[69] Morgan D.P., Kelley D.F. (2020). What Does the Transient Absorption Spectrum of CdSe Quantum Dots Measure?. J. Phys. Chem. C.

[70] Hunsche S., Dekorsy T., Klimov V., Kurz H. (1996). Ultrafast Dynamics of Carrier-Induced Absorption Changes in Highly-Excited CdSe Nanocrystals. Appl. Phys. B Laser Opt..

[71] Wu K., Zhu H., Liu Z., Rodríguez-Córdoba W., Lian T. (2012). Ultrafast Charge Separation and Long-Lived Charge Separated State in Photocatalytic CdS-Pt Nanorod Heterostructures. J. Am. Chem. Soc..

[72] Jasrasaria D., Philbin J.P., Yan C., Weinberg D., Alivisatos A.P., Rabani E. (2020). Sub-Bandgap Photoinduced Transient Absorption Features in CdSe Nanostructures: The Role of Trapped Holes. J. Phys. Chem. C.

[73] Reiss P., Protière M., Li L. (2009). Core/Shell Semiconductor Nanocrystals. Small.

[74] Zavelani-Rossi M., Lupo M.G., Tassone F., Manna L., Lanzani G. (2010). Suppression of Biexciton Auger Recombination in CdSe/CdS Dot/Rods: Role of the Electronic Structure in the Carrier Dynamics. Nano Lett..

[75] Smith E.R., Luther J.M., Johnson J.C. (2011). Ultrafast Electronic Delocalization in CdSe/CdS Quantum Rod Heterostructures. Nano Lett..

[76] Steiner D., Dorfs D., Banin U., Della Sala F., Manna L., Millo O. (2008). Determination of Band Offsets in Heterostructured Colloidal Nanorods Using Scanning Tunneling Spectroscopy. Nano Lett..

[77] She C., Demortière A., Shevchenko E.V., Pelton M. (2011). Using Shape to Control Photoluminescence from CdSe/CdS Core/Shell Nanorods. J. Phys. Chem. Lett..

[78] Sift A., Sala F.D., Menagen G., Banin U. (2009). Multiexciton Engineering in Seeded Core/Shell Nanorods: Transfer from Type-I to Quasi-Type-Ll Regimes. Nano Lett..

[79] Rainò G., Stöferle T., Moreels I., Gomes R., Kamal J.S., Hens Z., Mahrt R.F. (2011). Probing the Wave Function Delocalization in CdSe/CdS Dot-in-Rod Nanocrystals by Time- and Temperature-Resolved Spectroscopy. ACS Nano.

[80] Eshet H., Grünwald M., Rabani E. (2013). The Electronic Structure of CdSe/CdS Core/Shell Seeded Nanorods: Type-I or Quasi-Type-II?. Nano Lett..

[81] Wu K., Hill L.J., Chen J., McBride J.R., Pavlopolous N.G., Richey N.E., Pyun J., Lian T. (2015). Universal Length Dependence of Rod-to-Seed Exciton Localization Efficiency in Type I and Quasi-Type II CdSe@CdS Nanorods. ACS Nano.

[82] Wang L., Nonaka K., Okuhata T., Katayama T., Tamai N. (2018). Quasi-Type II Carrier Distribution in CdSe/CdS Core/Shell Quantum Dots with Type I Band Alignment. J. Phys. Chem. C.

[83] Rosner T., Pavlopoulos N.G., Shoyhet H., Micheel M., Wächtler M., Adir N., Amirav L. (2022). The Other Dimension—Tuning Hole Extraction via Nanorod Width. Nanomaterials.

[84] Utterback J.K., Grennell A.N., Wilker M.B., Pearce O.M., Eaves J.D., Dukovic G. (2016). Observation of Trapped-Hole Diffusion on the Surfaces of CdS Nanorods. Nat. Chem..

[85] Robinson R.D., Sadtler B., Demchenko D.O., Erdonmez C.K., Wang L.-W., Alivisatos A.P. (2007). Spontaneous Superlattice Formation in Nanorods Through Partial Cation Exchange. Science.

[86] Wu K., Rodríguez-Córdoba W., Lian T. (2014). Exciton Localization and Dissociation Dynamics in CdS and CdS–Pt Quantum Confined Nanorods: Effect of Nonuniform Rod Diameters. J. Phys. Chem. B.

[87] Utterback J.K., Hamby H., Pearce O.M., Eaves J.D., Dukovic G. (2018). Trapped-Hole Diffusion in Photoexcited CdSe Nanorods. J. Phys. Chem. C.

[88] Utterback J.K., Ruzicka J.L., Hamby H., Eaves J.D., Dukovic G. (2019). Temperature-Dependent Transient Absorption Spectroscopy Elucidates Trapped-Hole Dynamics in CdS and CdSe Nanorods. J. Phys. Chem. Lett..

[89] Kuno M., Fromm D.P., Hamann H.F., Gallagher A., Nesbitt D.J. (2000). Nonexponential “Blinking” Kinetics of Single CdSe Quantum Dots: A Universal Power Law Behavior. J. Chem. Phys..

[90] Grennell A.N., Utterback J.K., Pearce O.M., Wilker M.B., Dukovic G. (2017). Relationships between Exciton Dissociation and Slow Recombination within ZnSe/CdS and CdSe/CdS Dot-in-Rod Heterostructures. Nano Lett..

[91] Talapin D.V., Nelson J.H., Shevchenko E.V., Aloni S., Sadtler B., Alivisatos A.P. (2007). Seeded Growth of Highly Luminescent CdSe/CdS Nanoheterostructures with Rod and Tetrapod Morphologies. Nano Lett..

[92] Borys N.J., Walter M.J., Huang J., Talapin D.V., Lupton J.M. (2010). The Role of Particle Morphology in Interfacial Energy Transfer in CdSe/CdS Heterostructure Nanocrystals. Science.

[93] Wu K., Rodríguez-Córdoba W.E., Liu Z., Zhu H., Lian T. (2013). Beyond Band Alignment: Hole Localization Driven Formation of Three Spatially Separated Long-Lived Exciton States in CdSe/CdS Nanorods. ACS Nano.

[94] Ben-Shahar Y., Scotognella F., Waiskopf N., Kriegel I., Conte S.D., Cerullo G., Banin U. (2015). Effect of Surface Coating on the Photocatalytic Function of Hybrid CdS-Au Nanorods. Small.

[95] O’Connor T., Panov M.S., Mereshchenko A., Tarnovsky A.N., Lorek R., Perera D., Diederich G., Lambright S., Moroz P., Zamkov M. (2012). The Effect of the Charge-Separating Interface on Exciton Dynamics in Photocatalytic Colloidal Heteronanocrystals. ACS Nano.

[96] Ben-Shahar Y., Scotognella F., Kriegel I., Moretti L., Cerullo G., Rabani E., Banin U. (2016). Optimal Metal Domain Size for Photocatalysis with Hybrid Semiconductor-Metal Nanorods. Nat. Commun..

[97] Liu Y., Yang W., Chen Q., Cullen D.A., Xie Z., Lian T. (2022). Pt Particle Size Affects Both the Charge Separation and Water Reduction Efficiencies of CdS–Pt Nanorod Photocatalysts for Light Driven H_2_ Generation. J. Am. Chem. Soc..

[98] Kalisman P., Houben L., Aronovitch E., Kauffmann Y., Bar-Sadan M., Amirav L. (2015). The Golden Gate to Photocatalytic Hydrogen Production. J. Mater. Chem. A.

[99] Huang J., Huang Z., Yang Y., Zhu H., Lian T. (2010). Multiple Exciton Dissociation in CdSe Quantum Dots by Ultrafast Electron Transfer to Adsorbed Methylene Blue. J. Am. Chem. Soc..

[100] Benndorf S., Schleusener A., Müller R., Micheel M., Baruah R., Dellith J., Undisz A., Neumann C., Turchanin A., Leopold K. (2023). Covalent Functionalization of CdSe Quantum Dot Films with Molecular [FeFe] Hydrogenase Mimics for Light-Driven Hydrogen Evolution. ACS Appl Mater Interfaces.

[101] Matylitsky V.V., Dworak L., Breus V.V., Basché T., Wachtveitl J. (2009). Ultrafast Charge Separation in Multiexcited CdSe Quantum Dots Mediated by Adsorbed Electron Acceptors. J. Am. Chem. Soc..

[102] Jiang Z.-J., Kelley D.F. (2011). Hot and Relaxed Electron Transfer from the CdSe Core and Core/Shell Nanorods. J. Phys. Chem. C.

[103] Schleusener A., Micheel M., Benndorf S., Rettenmayr M., Weigand W., Wächtler M. (2021). Ultrafast Electron Transfer from CdSe Quantum Dots to an [FeFe]-Hydrogenase Mimic. J. Phys. Chem. Lett..

[104] Thomas A., Sandeep K., Somasundaran S.M., Thomas K.G. (2018). How Trap States Affect Charge Carrier Dynamics of CdSe and InP Quantum Dots: Visualization through Complexation with Viologen. ACS Energy Lett..

[105] Tseng H.-W., Wilker M.B., Damrauer N.H., Dukovic G. (2013). Charge Transfer Dynamics between Photoexcited CdS Nanorods and Mononuclear Ru Water-Oxidation Catalysts. J. Am. Chem. Soc..

[106] Huang J., Huang Z., Jin S., Lian T. (2008). Exciton Dissociation in CdSe Quantum Dots by Hole Transfer to Phenothiazine. J. Phys. Chem. C.

[107] Lian S., Weinberg D.J., Harris R.D., Kodaimati M.S., Weiss E.A. (2016). Subpicosecond Photoinduced Hole Transfer from a CdS Quantum Dot to a Molecular Acceptor Bound Through an Exciton-Delocalizing Ligand. ACS Nano.

[108] Pearce O.M., Duncan J.S., Damrauer N.H., Dukovic G. (2018). Ultrafast Hole Transfer from CdS Quantum Dots to a Water Oxidation Catalyst. J. Phys. Chem. C.

[109] Berr M.J., Vaneski A., Mauser C., Fischbach S., Susha A.S., Rogach A.L., Jäckel F., Feldmann J. (2012). Delayed Photoelectron Transfer in Pt-Decorated CdS Nanorods under Hydrogen Generation Conditions. Small.

[110] Beckwith J.S., Lang B., Grilj J., Vauthey E. (2019). Ion-Pair Dynamics upon Photoinduced Electron Transfer Monitored by Pump-Pump-Probe Spectroscopy. J. Phys. Chem. Lett..

[111] Pagès S., Lang B., Vauthey E. (2006). Ultrafast Excited State Dynamics of the Perylene Radical Cation Generated upon Bimolecular Photoinduced Electron Transfer Reaction. J. Phys. Chem. A.

[112] Kuss-Petermann M., Wenger O.S. (2017). Pump-Pump-Probe Spectroscopy of a Molecular Triad Monitoring Detrimental Processes for Photoinduced Charge Accumulation. Helv. Chim. Acta.

[113] Tran T.T., Pino T., Ha-Thi M.H. (2019). Watching Intermolecular Light-Induced Charge Accumulation on Naphthalene Diimide by Tris(Bipyridyl)Ruthenium(II) Photosensitizer. J. Phys. Chem. C.

[114] Ha-Thi M.H., Pham V.T., Pino T., Maslova V., Quaranta A., Lefumeux C., Leibl W., Aukauloo A. (2018). Photoinduced Electron Transfer in a Molecular Dyad by Nanosecond Pump-Pump-Probe Spectroscopy. Photochem. Photobiol. Sci..

[115] Neumann S., Kerzig C., Wenger O.S. (2019). Quantitative Insights into Charge-Separated States from One- and Two-Pulse Laser Experiments Relevant for Artificial Photosynthesis. Chem. Sci..

[116] Wang J., Ding T., Leng J., Jin S., Wu K. (2018). “Intact” Carrier Doping by Pump-Pump-Probe Spectroscopy in Combination with Interfacial Charge Transfer: A Case Study of CsPbBr3 Nanocrystals. J. Phys. Chem. Lett..

[117] Wang J., Ding T., Wu K. (2020). Coulomb Barrier for Sequential Two-Electron Transfer in a Nanoengineered Photocatalyst. J. Am. Chem. Soc..

[118] Scheidt R.A., Samu G.F., Janáky C., Kamat P.V. (2018). Modulation of Charge Recombination in CsPbBr_3_ Perovskite Films with Electrochemical Bias. J. Am. Chem. Soc..

[119] Qin W., Guyot-Sionnest P. (2012). Evidence for the Role of Holes in Blinking: Negative and Oxidized CdSe/CdS Dots. ACS Nano.

[120] Kambhampati P. (2012). Multiexcitons in Semiconductor Nanocrystals: A Platform for Optoelectronics at High Carrier Concentration. J. Phys. Chem. Lett..

[121] Klimov V.I., McGuire J.A., Schaller R.D., Rupasov V.I. (2008). Scaling of Multiexciton Lifetimes in Semiconductor Nanocrystals. Phys. Rev. B Condens. Matter Mater. Phys..

[122] Zhu H., Yang Y., Lian T. (2013). Multiexciton Annihilation and Dissociation in Quantum Confined Semiconductor Nanocrystals. Acc. Chem. Res..

[123] Schaller R.D., Sykora M., Jeong S., Klimov V.I. (2006). High-Efficiency Carrier Multiplication and Ultrafast Charge Separation in Semiconductor Nanocrystals Studied via Time-Resolved Photoluminescence. J. Phys. Chem. B.

[124] Schaller R.D., Klimov V.I. (2004). High Efficiency Carrier Multiplication in PbSe Nanocrystals: Implications for Solar Energy Conversion. Phys. Rev. Lett..

[125] Ellingson R.J., Beard M.C., Johnson J.C., Yu P., Micic O.I., Nozik A.J., Shabaev A., Efros A.L. (2005). Highly Efficient Multiple Exciton Generation in Colloidal PbSe and PbS Quantum Dots. Nano Lett..

[126] Ben-Shahar Y., Philbin J.P., Scotognella F., Ganzer L., Cerullo G., Rabani E., Banin U. (2018). Charge Carrier Dynamics in Photocatalytic Hybrid Semiconductor-Metal Nanorods: Crossover from Auger Recombination to Charge Transfer. Nano Lett..

[127] Lupo M.G., Sala F.D., Carbone L., Zavelani-Rossi M., Fiore A., Lüer L., Polli D., Cingolani R., Manna L., Lanzani G. (2008). Ultrafast Electron-Hole Dynamics in Core/Shell CdSe/CdS Dot/Rod Nanocrystals. Nano Lett..

[128] Zhu H., Song N., Rodríguez-Córdoba W., Lian T. (2012). Wave Function Engineering for Efficient Extraction of up to Nineteen Electrons from One CdSe/CdS Quasi-Type II Quantum Dot. J. Am. Chem. Soc..

[129] Yan C., Weinberg D., Jasrasaria D., Kolaczkowski M.A., Liu Z., Philbin J.P., Balan A.D., Liu Y., Schwartzberg A.M., Rabani E. (2021). Uncovering the Role of Hole Traps in Promoting Hole Transfer from Multiexcitonic Quantum Dots to Molecular Acceptors. ACS Nano.

[130] Zhu H., Lian T. (2012). Enhanced Multiple Exciton Dissociation from CdSe Quantum Rods: The Effect of Nanocrystal Shape. J. Am. Chem. Soc..

[131] Young R.M., Jensen S.C., Edme K., Wu Y., Krzyaniak M.D., Vermeulen N.A., Dale E.J., Stoddart J.F., Weiss E.A., Wasielewski M.R. (2016). Ultrafast Two-Electron Transfer in a CdS Quantum Dot–Extended-Viologen Cyclophane Complex. J. Am. Chem. Soc..

[132] Liu Y., Cullen D.A., Lian T. (2021). Slow Auger Recombination of Trapped Excitons Enables Efficient Multiple Electron Transfer in CdS–Pt Nanorod Heterostructures. J. Am. Chem. Soc..

